# Long-term outcomes of high-risk human papillomavirus infection support a long interval of cervical cancer screening

**DOI:** 10.1038/sj.bjc.6604262

**Published:** 2008-02-19

**Authors:** Y-K Huang, S-L You, C-C Yuan, Y-M Ke, J-M Cao, C-Y Liao, C-H Wu, C-S Hsu, K-F Huang, C-H Lu, N-F Twu, T-Y Chu

**Affiliations:** 1 Department of Obstetrics and Gynecology, Buddhist Tzu Chi General Hospital, Tzu Chi University, Hualien, Taiwan, ROC; 2 Graduate Institute of Clinical Medicine, Tzu Chi University, Hualien, Taiwan, ROC; 3 Genomics Research Center, Academia Sinica, Taipei, Taiwan, ROC; 4 Department of Obstetrics and Gynecology, Taipei Veterans General Hospital, National Yang-Ming University, Taiwan, ROC; 5 Division of Gynecologic Oncology, Department of Obstetrics and Gynecology, Taichung Veterans General Hospital, Taichung, Taiwan, ROC; 6 Department of Obstetrics and Gynecology, Ton-Yen General Hospital, Ju-Pei, Taiwan, ROC; 7 Department of Obstetrics and Gynecology, Mennonite Christian Hospital, Hualien, Taiwan, ROC; 8 Department of Obstetrics and Gynecology, Chung-Ho Memorial Hospital, Kaohsiung Medical University, Kaohsiung, Taiwan, ROC; 9 Department of Obstetrics and Gynecology, Taipei Medical University Municipal Wan Fang Hospital, Taipei, Taiwan, ROC; 10 Department of Obstetrics and Gynecology, Chi-Mei Medical Center, Tainan, Taiwan, ROC

**Keywords:** human papillomavirus, cervical cancer, long-term follow-up, absolute risk

## Abstract

Knowing that infection of high-risk human papillomavirus (HPV) causes virtually all cervical cancer (CC), the long-term outcomes of HPV infection, especially the absolute risk and time lapse of developing CC, are beyond the scope of ordinary follow-up study owing to ethical concerns. The present study followed the natural history and long-term outcomes of HPV infection in a cohort of women by national health insurance care and data linkage without additional disturbance. The status of cervical HPV infection was determined in 1708 healthy women, aged 20–90 (median 43), enrolled from 10 hospitals in seven cities around the island country of Taiwan. Records of consecutive Pap smear results and cancer reports of 108 cytology-negative, HPV-positive and 1202 cytology- and HPV-negative women with no prior record of CC or abnormal cervical cytology were retrospectively analysed for a duration of up to 75 months (median 61 months). The cumulative incidences of high-grade squamous intraepithelial lesion (HSIL) and *in situ*/invasive cancer in HPV-positive women were 5.6 and 3.7%, respectively, and those in HPV-negative women were 0.3 and 0%. After adjusting for other risk factors, HPV-positive subjects had 24.9 (95% CI: 7.0–108.3; *P*<0.0001) folds of risk of developing HSIL or above cervical neoplasia as compared to HPV-negative subjects, whereas risk for low-grade intraepithelial lesion and atypical squamous cytology was not increased. The study showed that women with a prevalent infection of high-risk HPV had a 4% cumulative risk for CC in 6 years, whereas those tested negative had little risk. The result supports an HPV test-orientated CC screening programme with intervals of at least 5 years.

The aetiological and universal roles of high-risk human papillomavirus (HPV) infection in cervical carcinogenesis have been well established. Primary and secondary preventive measures targeting on the vaccination and detection of HPV have emerged as effective public health strategies (Bulkmans *et al*, 2004; Kitchener *et al*, 2006; Mayrand *et al*, 2006; Wheeler, 2007). Before a wide application of these preventive measures can be realised, an accurate estimation of the long-term outcome of HPV infection is critically in demand. A major but rarely studied outcome measure is the absolute risk and sojourn time of developing cervical cancer (CC) or its precursor in the presence of HPV infection (Sherman *et al*, 2003). In the face of differences in host and environmental factors, as well as the public and private health-care formats, the natural history of HPV infection varied widely among different populations. A regional follow-up of HPV infection is therefore critically needed as the baseline epidemiological data for cost-effectiveness modelling and policy making (Dasbach *et al*, 2006; Newall *et al*, 2007; Wheeler, 2007).

Given the long and heterogeneous natural history of HPV infection and the lack of a robust animal model or culture system, studying the natural history of HPV infection typically requires a cohort of long-term follow-up that is both labour intensive and expensive. Following subjects beyond the precancer stage is ethically not allowed. An alternative way is to utilize the registry of medical records to follow subjects passively. Previous studies of passive follow-up tested HPV infection serologically and retrospectively (Hinkula *et al*, 2004; Peto *et al*, 2004; Korodi *et al*, 2005; Kjaer *et al*, 2006). In this prospective study, we conducted a standard HPV DNA test and Pap smear and followed cervical neoplasia by medical records linkage in a cohort of subjects who were cared for by the national health insurance programme. A 6-year risk and sojourn time of occurrence of cervical neoplasia in relation to HPV infection in a real world were unveiled.

## MATERIALS AND METHODS

### Study subjects and database linkage

**Figure 1:**
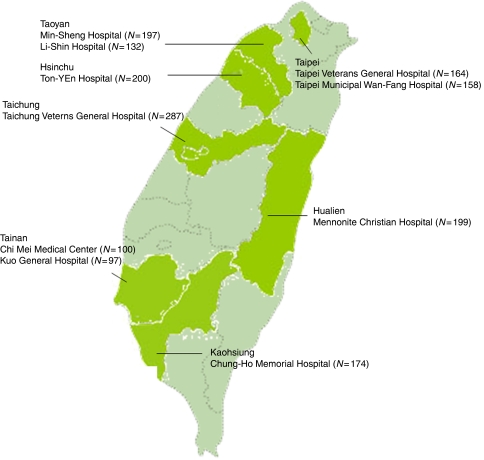
Locations of study sites. The number of study sites was proportional to the population density in the five major areas: five in the north, four in the south and one each in the middle west and the east part of Taiwan.

On Mother's Day (May 13) in 2000, a campaign of CC screening including a single-visit Pap test and Hybrid Capture 2 (HC2; Digene, Gaitherburg, MD, USA) HPV DNA testing was implemented for mothers who had not been screened for at least 1 year at 10 general hospitals from seven major cities around Taiwan: two in Taipei metropolitan area, three in the north, one in the central west, three in the southern west and one in the east of the island (Figure 1). At the visit, standard demographic data, cancer-related history and history relating to Pap smear interpretation were recorded. The latter included menstrual and pregnant history, contraceptive method, past or present history of cervical neoplasia and previous history of hysterectomy or radiation. Part of the cervical scraping was sent for HPV test, and the subjects were informed of the test results. A total of 1708 women who agreed to the screening activity and had a successful HPV DNA test detected by HC2 were enrolled. All subjects were cared for by the National Health Insurance system, which provides comprehensive health services, including preventive and medical services, prescription drugs and others, with good accessibility at reasonable cost (Lu and Hsiao, 2003). To reveal the outcomes of HPV infection in the real world, enrolled subjects were left undisturbed and the end points of cervical neoplasia were collected through the national database of Pap smear and cancer registry.

**Figure 2:**
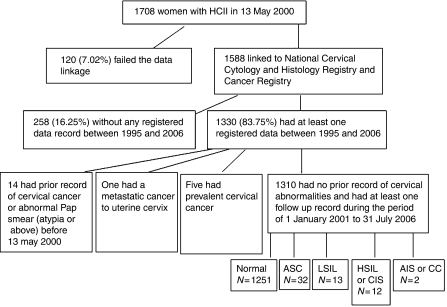
A schematic overview of the data linkage and follow-up outcomes. After exclusion of ineligible subjects, 1310 women fulfilled the follow-up criteria and the outcome classification was given.

A schematic overview of the study design and case outcomes was shown in Figure 2. One hundred and twenty subjects refused the follow-up and did not provide their personal identification number (ID). The remaining 1588 subjects were passively followed by linking their ID to the Taiwan Cervical Cytology Screening and Histology Database and to the Taiwan Cancer Registry in August 2006. This data linking analysis was approved by the Bureau of Health Promotion, Department of Health, Taiwan. Among the 1588 subjects, 288 having no record of Pap smear or cancer registry data during the follow-up period were excluded. Also excluded were 14 subjects who had records of abnormal Pap smear test results (including Atypia or more severe results) or CC prior to 13 May 2000, and five subjects having CC detected on 13 May 2000. Pap smear and cancer registry data of the remaining 1310 eligible subjects were analysed. Their median age was 43 (ranged 23–80) years.

### Pap smear follow-up

The National Health Insurance of Taiwan provides annual Pap smear screening for women aged 30 or above since 1995 (Koong *et al*, 2006). The Pap smear screening programme included a quality control monitoring and training system covering the physician, public health nurses, cytologists and cytotechnicians. The results of Pap smears and cervical biopsies were recorded in the National Cervical Cytology and Histology Registry as a comprehensive and confidential database. A 3-year screening rate increased from 14.5% in 1995 to 53.8% in 2005 (Department of Health Executive Yuan Taiwan, 2005). The cytology report followed the Bethesda System and shifted to its new version in 2002 (Solomon *et al*, 2002). In the analysis, we reclassified the cytology results into Normal (including ‘normal’ and ‘benign reactive change’), Atypia (including ‘atypical squamous cells of undetermined significance’ or ‘atypical glandular cells of undetermined significance’), low grade squamous intraepithelial lesion (LSIL), high-grade squamous intraepithelial lesion (HSIL) (excluding carcinoma *in situ* (CIS)) and CIS/adenocarcinoma *in situ* (AIS)/CC. The most severe cytology results occurring during the follow-up period were counted, and the lag time was counted between the date of enrolment and the date of occurrence of the most severe abnormal cytology.

### Follow-up by cancer registry

The Taiwan Cancer Registry was established in 1970. The data were input from the medical delivery system consisting of one cancer hospital, 18 medical centres and hundreds of regional and district general hospitals. In 1995, 81% of the reported cancers were confirmed by pathological examinations, 13% by imaging diagnosis and 6% by other methods (Chen *et al*, 2002). Since the implementation of the National Health Insurance in 1995, 97 and 99% of the total population have been enrolled by 2001 (Lu and Hsiao, 2003) and 2007, respectively. The reporting rate of the Taiwan Cancer Registry is one of the highest in the world. The ID of the eligible subjects was linked to the registry and looked for the occurrence of CC with the ICD9 disease code of 180 during the period between May 2000 and August 2006. Cervical cancer records before May 2000 were also surveyed to exclude the ineligible subjects. There were nine cancers on record between May 2000 and August 2006. Among them, five cancers occurred on enrolment, which were not possibly a consequence of the recent HPV infection, and were excluded. There was one metastatic cancer from the colon, which was also excluded. The remaining three were two CIS and one invasive cancer of the uterine cervix, which occurred at the 28th, 32nd and 34th months, respectively, after enrolment.

### HPV detection

Hybrid Capture 2 high-risk HPV DNA test was conducted. Cervical scrapings were collected by using the cytobrush and transfer medium provided by the manufacturer. The same cytobrush was used for Pap smear before dipping into the transfer medium for HPV test. Hybrid Capture 2 used RNA probes specific for 13 cancer-associated HPV types. The DNA–RNA hybrids were captured and detected by reporter molecule-labelled antibodies as described before (Sun *et al*, 2001). Coefficients of variation of the triplicate tests of both the positive (50 fg or 5000 copies of HPV-16 DNA) and the negative (100 ng of carrier DNA) controls (*n*=3) were less than 5%, respectively, and the ratio of positive mean to negative mean was more than 3.0.

Human papillomavirus genotyping in HC2-positive cervical scrapings was determined by consensus PCR and reverse blot hybridisation as previously described (Huang *et al*, 2006; Lin *et al*, 2007). Briefly, the SPF1/GP6 consensus primers were used to amplify a fragment of approximately 184 bp in the L1 open reading frame. The PCR products were then hybridised with an Easychip HPV Blot (King Car, Yilan, Taiwan) that included 39 types of HPV (types 6, 11, 16, 18, 26, 31, 32, 33, 35, 37, 39, 42, 43, 44, 45, 51, 52, 53, 54, 55, 56, 58, 59, 61, 62, 66, 67, 68, 69, 70, 72, 74, 82, CP8061, CP8304, L1AE5, MM4, MM7 and MM8) of oligonucleotide probes and were visualised by alkaline phosphatase conjugation and biotinylated antibodies (Huang *et al*, 2006; Lai *et al*, 2007).

### Statistical analysis

STATA version 8 was used for data analysis. *χ*^2^ test was used to assess the association of categorical variables and Student's *t*-test was used to assess continuous variables. Kaplan–Meier plots were used to compare the nonoccurrence of cervical neoplasia of participants with or without HPV infection. Log-rank test was used for comparison of the nonoccurrence curves between HPV-positive and HPV-negative subjects. By use of the Cox proportional hazards model, multivariate analysis was performed to determine the independent impact of HPV infection on survival. Failure events were defined as the occurrences of (1) Atypia, (2) LSIL and (3) HSIL or above neoplasia. For all statistical tests, the level of significance was set at 0.05.

## RESULTS

All the subjects studied had a normal Pap smear result at enrolment on 13 May 2000 and at least one Pap smear or cancer registry entry data during the follow-up period till 31 August 2006. As shown in Table 1, the duration of follow-up did not differ between HPV-positive and HPV-negative subjects (57.80±0.39 *vs* 59.52±1.21 months) (mean±s.e.m.). As expected, HPV-positive subjects had a slightly shorter follow-up interval (24.47±1.45 months) than the HPV-negative (27.38±0.43 months) subjects (*P*=0.06). The average number of follow-up was slightly higher in HPV-positive groups than in HPV-negative groups (3.15±0.23 *vs* 2.79±0.04). When stratified by follow-up outcomes, the follow-up intervals did not differ between the two groups (Table 1).

In the screened population, which had a median age of 43 years (ranging from 20 to 90), the prevalence of HPV infection was 8.24% (108/1310). The rate tended to increase with age, from 8.08% in age 30–40 years to 10.0% in age over 60 years, but without statistical significance. Among different geographic regions, participants living in Taoyan and Hsinchu had a slightly but insignificantly higher HPV prevalence (9.64%) as compared to other regions (6.37–8.66%) (Table 2).

**Figure 3:**
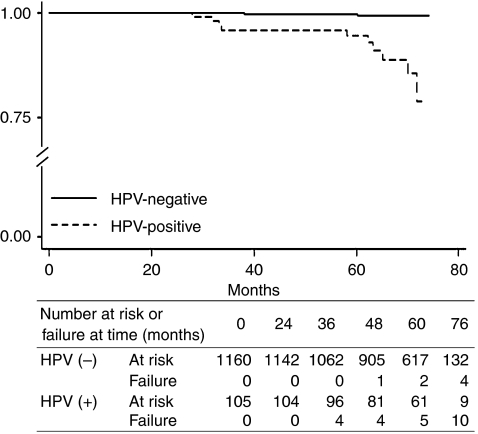
Kaplan–Meier estimates of HSIL or above outcome in HPV-positive and HPV-negative subjects. Failure was defined as the occurrence of HSIL or above cervical neoplasia. The adjusted hazard ratio was 24.9 (7.01–108.27) (*P*<0.0001). HPV=human papillomavirus; HSIL=high-grade squamous intraepithelial lesion.

The records of consecutive Pap smear results and cancer reports of 108 cytology-negative, HPV-positive and 1202 cytology- and HPV-negative women were followed for a median duration of 61 months. In the meanwhile, there were five prevalent cases of CC, who were all HPV-positive, being excluded from the follow-up. The status of HPV infection on enrolment mattered greatly to the outcomes of follow-up. In HPV-positive subjects (*n*=108), 12% developed cervical neoplasia, whereas only 3.8% of HPV-negative subjects (*n*=1202) did so. The major difference was the occurrence rate of HSIL or above neoplasia, which was 9.25% in the HPV-positive group and 0.14% in the HPV-negative group. All the four subjects with follow-up outcomes of CIS (*n*=2), AIS or CC were HPV-positive on enrolment (Table 3). When comparing different follow-up outcomes, the HPV prevalence at enrolment was significantly higher in the CIS/AIS/CC outcome group (100%) than in the HSIL outcome (60.0%), Atypia outcome (9.4%), Normal outcome (7.6%) and LSIL outcome (0%) groups (*P*<0.0001, Table 3). As shown in Figure 3, during a maximal follow-up of 74 months, the occurrence of HSIL or above cervical neoplasia was significantly higher in the HPV-positive subjects than in the HPV-negative subjects, with an adjusted hazard ratio of 24.9 (7.01–108.27) (*P*<0.0001). Table 4 summarises the incidence and hazard ratio of different follow-up outcomes. Compared to HPV-negative subjects, the incidence of HSIL or above neoplasia in HPV-positive subjects was significantly higher (0.0194 *vs* 0.0007), but this was not true in the Atypia and LSIL groups.

Figure 3 shows the Kaplan–Meier estimates of the occurrence of HSIL or above cervical neoplasia during the follow-up. The adjusted hazard ratio for HPV-positive *vs* HPV-negative subjects was 24.9 (7.01–108.27) (*P*<0.0001). The cumulative risk for HSIL or above cervical neoplasia in HPV-positive subjects was 3.88% in 4 years, 4.88% in 5 years and 10.0% in 6 years. For HPV-negative subjects, the cumulative risks were 0.086, 0.26 and 0.34% in 4, 5 and 6 years, respectively.

Table 5 shows the follow-up results of subjects infected with different HPV types. Although 27 different high-risk and low-risk HPV types were found in subjects with Normal outcome, only limited high-risk types such as types 16, 18, 31, 51, 58, 59 and 70 were found in subjects with outcomes of HSIL or above cervical neoplasia. Owing to small sample sizes in each HPV type stratum, no significant differences were noted among different HPV types or groups.

## DISCUSSION

A nonintervention, passive follow-up of HPV-infected subjects was conducted in this study as a way to explore the natural history of the infection in the real world of a universal health insurance care system. The absolute risks of the occurrence of cervical neoplasia were obtained in infected and noninfected subjects. The prevalence of high-risk HPV infection determined by HC2 (8.2%) was similar to that in other reports from the same population (Liaw *et al*, 1995; Department of Health Executive Yuan Taiwan, 2005; Sun *et al*, 2005). HPV prevalence did not differ among the seven areas studied in this island country. Besides, the results of Pap smear screening including ages (median 43 years, ranged from 20 to 90) and distributions of abnormal cytology (2.4, 1.0 and 0.8% of Atypia, LSIL and HSIL or above, respectively) did not differ from those reported in the National Registry (Department of Health Executive Yuan Taiwan, 2005), indicating a good representation of this cohort to this small island country.

Passive follow-up of cervical neoplasia requires a well-established public or private health-care system with long-term and consistent coverage of the target population (Castle *et al*, 2002) and comprehensive databases. The possible limitations of this study include uncontrolled follow-up intervals, low coverage rate and insufficient disease assessment. Although an annual screening policy was conducted in this country, the 3-year coverage rate was 55% and the screening interval of this cohort was 24 months, which was shorter than other cohorts conducted in the UK (the Manchester cohort) (Peto *et al*, 2004) and Denmark (Kjaer *et al*, 2006) where screening intervals were typically 3–5 years. As an augmentation to this limitation, the national cancer registry database, which records all cancer cases from the diagnosing hospitals, was also applied to identify prevalent and incident CIS and CC that may be missed by Pap smear screening or diagnosed without screening. As subjects were informed of the result of the HPV test, a positive test result may motivate more frequent, regular follow-up visits that may be biased towards a higher detection rate of cervical neoplasia. The follow-up intervals in the HPV-positive subjects were somewhat shorter than in the HPV-negative subjects (24.5 *vs* 27.4 months, *P*=0.06). However, after exclusion of subjects with neoplastic outcomes, those with Normal outcome had similar follow-up intervals in the HPV-positive and HPV-negative groups (25.3 *vs* 27.9 months, *P*=0.1).

Previous long-term follow-up studies of the outcomes of HPV infection were limited (Rozendaal *et al*, 2000; Schlecht *et al*, 2001; Castle *et al*, 2002; Peto *et al*, 2004; Kjaer *et al*, 2006). Early studies used the insensitive cytology but not DNA test as the diagnostic criteria (Nash *et al*, 1987; Syrjanen *et al*, 1992). Some studies were focused on young women whose HSIL were less likely to progress to more severe neoplasia (Koutsky *et al*, 1992; Woodman *et al*, 2001). This study aimed to elucidate the long-term outcome of the population at risk for CC. Mature women were followed for the occurrence of CC and its precursor for up to 75 months. The 4-, 5- and 6-year cumulative incidences of HSIL or above neoplasia were 3.9, 4.9 and 10%, respectively, in HPV-positive women, as compared to 0.09, 0.26 and 0.34% in the HPV-negative women. A trend of increasing cumulative risk along the follow-up period beyond 4 years was noted, indicating the need for longer follow-up for the severe outcomes. A British cohort of passive follow-up also showed rising cumulative risk of CIN3 or cancer in HPV-positive women, reaching 28% by 14 years (1). HPV-positive women followed by the Danish Pathology Data Bank showed a 10-year risk for CIN3 or cancer of 13.6 and 21% for young (22–32 years) and old (40–50 years) women, respectively. As a comparison, a lower 5-year cumulative incidence of HSIL or above neoplasia was noted in the Portland cohort (Castle *et al*, 2002), and a much higher figure (about 25 *vs* 5%) was noted in the Brazil cohort (Schlecht *et al*, 2001). Although the status of risk factors other than HPV infection, such as the environmental (such as smoking and other genital infections) (Koskela *et al*, 2000; Smith *et al*, 2002) and genetic susceptibility (Caucasian *vs* Chinese) to cervical carcinogenesis can be different among cohorts, the accessibility and extent of medical interventions, such as Pap smear, cervical biopsy and local treatments, may be the key differences among the settings. The subjects in this cohort were completely undisturbed after a single visit for Pap smear and HPV testing. The results thus figure the real natural history of HPV infection in the population in Taiwan.

In contrast to the occurrence of HSIL and cancer, which was highly associated with HPV infection at enrolment, in this study and that of the Manchester cohort (Peto *et al*, 2004), the occurrence of LSIL was found to be unrelated to the initial HPV status. LSIL is known to be an immediate and transient disease of HPV infection. Given a follow-up interval of 24 months, most of the HPV-related LSIL that was missed by cytology at enrolment may have been regressed at the first follow-up. Meanwhile, none of the subjects tested negative for both cytology and high-risk HPV on enrolment developed CIS or invasive cancer during the 6-year follow-up. Women who are HPV-negative bear little risk for CC in this study. This supports a focused cytology screening of HPV-infected women. In the current socio-economic setting in Taiwan, an HPV test-orientated screening is suggested. Only HPV-positive women have to be screened annually. Human papillomavirus-negative women may safely extend their screening interval to 5 years at least.

## Figures and Tables

**Table 1:** Durations, intervals and outcomes of Pap smear follow-up in HPV-positive and HPV-negative subjects

	**HPV-negative subjects**			**HPV-positive subjects**			
**Follow-up outcome^a^**	**No**	**FU duration^b^ (month)**	**FU interval (month)**	**No**	**FU duration^b^ (month)**	**FU interval (month)**	***P*-value^c^**
Normal	1156	58.06±0.39	27.83±0.44	95	59.57±1.27	25.25±1.46	*P*=0.10
Atypia	29	50.53±3.42	17.93±2.41	2	55.31±9.46	9.76±1.41	*P*=0.01
LSIL	13	51.99±3.76	11.51±1.14	0	—	—	—
HSIL or above^d^	4	55.08±6.32	17.53±4.46	7	60.59±4.90	20.15±8.30	*P*=0.83
All	1202	57.80±0.39	27.38±0.43	105	59.52±1.21	24.47±1.45	*P*=0.06

Abbreviations: HPV=human papillomavirus; HSIL=high-grade squamous intraepithelial lesion.

a See Materials and Methods for the definitions.

b In outcomes other than Normal, the follow-up (FU) duration equals to the time to occurrence.

c Comparison of follow-up intervals between HPV-positive and HPV-negative subjects. *P*-value for Student’s *t*-test.

d The three cancer victims identified by Cancer Registry were not included.

**Table 2:** Status of high-risk HPV infection on enrolment and outcome of follow-up

	**Total subject (*n*=1310)**	**HPV-positive (*n*=108)**	**HPV prevalence % (95% CI)**	***χ*^2^ (*P*-value)**
*Age category*				
Age<30	55	5	9.09 (3.02–19.95)	*χ*^2^=0.22 (*P*=0.99)
30⩽age<40	421	34	8.08 (5.66–11.1)	
40⩽age<50	503	40	7.95 (5.74–10.67)	
50⩽age<60	214	17	7.94 (4.69–12.41)	
60⩽age	100	10	10.00 (4.90–17.62)	
Missing	17	2	11.76 (1.46–36.44)	
				
*Area of residence*				
Taipei	277	24	8.66 (5.63–12.61)	*χ*^2^=2.56 (*P*=0.63)
Taoyuan and Hsinchu	394	38	9.64 (6.91–12.24)	
Taichung	215	18	8.37 (5.03–12.91)	
Tainan and Kaohsiung	267	18	6.74 (4.04–10.44)	
Hualien	157	10	6.37 (3.1–11.4)	

Abbreviations: HPV=human papillomavirus.

**Table 3:** Outcomes of follow-up and the HPV status on enrolment

	**Total**	**HPV (−) *n*=1202**	**HPV (+) *n*=108**	**HPV prevalence at enrolment (%)**	
*Follow-up results* a					
Normal	1251	1156 (96.2%)	95 (88.0%)	7.6 (6.9–13.0)	*χ*^2^=81.85
Atypia	32	29 (2.4%)	3 (2.8%)	9.4 (2.0–25.0)	(*P*<0.0001)
LSIL	13	13 (1.1%)	0 (0.0%)	0.0 (0.0–24.7)	
HSIL	10	4 (0.3%)	6 (5.6%)	60.0 (26.2–87.8)	
CIS/AIS/CC	4	0 (0.0%)	4 (3.7%)	100.0 (39.8–100.0)	
All	1310	1202 (100%)	108 (100%)	8.2 (6.81–9.9)	

Abbreviations: CC=cervical cancer; HPV=human papillomavirus; HSIL=high-grade squamous intraepithelial lesion.

a See Materials and Methods for the definition of the categories.

**Table 4:** Incidence rate and hazard ratio of cervical neoplasia in relation to the HPV status at enrolment

	**HSIL or above^a^**			**Atypia^a^**			**LSIL^a^**		
	**HPV (−)**	**HPV (+)**	**Total**	**HPV (−)**	**HPV (+)**	**Total**	**HPV (−)**	**HPV (+)**	**Total**
Incidence case	4	10	14	29	2	35	13	0	13
Follow-up time (person-year)	5623	515	6138	5732	498	6230	5660	473	6133
6-year cumulative incidence	0.0034	0.1000	0.0111	0.0247	0.0310	0.0277	0.0111	—	0.0111
Incidence rate	0.0007	0.0194	0.0022	0.0051	0.0060	0.0051	0.0023	—	0.0021
Incidence rate ratio (95% CI)		27.30			1.19			—	
		(7.87–119.23)			(0.23–3.84)				
Hazard ratio (95% CI)	1.0	26.29		1.0	1.27				
		(8.24–83.86)^*^			(0.39–4.17)				
Adjusted hazard ratio^b^ (95% CI)	1.0	24.89		1.0	0.85			—	
		(7.01–108.27)^*^			(0.20–3.56)				
*P*-value for log-rank test		*P*<0.0001			*P*=0.69				

Abbreviations: HPV=human papillomavirus; HSIL=high-grade squamous intraepithelial lesion.

^*^P<0.0001.

a See Materials and Methods for the definitions.

b Age, area of residence and follow-up interval were adjusted using the Cox proportion hazard model.

**Table 5:** The distribution of HPV genotypes in different follow-up outcomes among the HC2-positive subjects

	**Pap normal subjects**	**Atypia**	**LSIL**	**HSIL or above**
**HPV on enrolment**	**No. (%)**	**No. (%)**	**No. (%)**	**No. (%)**
Negative	1156 (92.41)	29 (90.63)	13 (100.0)	4 (28.57)
Positive	95 (7.59)	3 (9.38)	0 (0.00)	10 (71.43)
				
*HPV type*				
6	1 (0.87)	1 (12.5)	—	—
11	3 (2.61)	1 (12.5)	—	—
16	10 (8.7)	—	—	4 (28.57)
18	9 (7.83)	1 (12.5)	—	2 (14.29)
31	2 (1.74)	1 (12.5)	—	2 (14.29)
32	1 (0.87)	—	—	—
33	4 (3.48)	—	—	—
35	1 (0.87)	—	—	—
39	3 (2.61)	—	—	—
44	1 (0.87)	—	—	—
45	—	1 (12.5)	—	—
51	3 (2.61)	1 (12.5)	—	1 (7.14)
52	10 (8.7)	1 (12.5)	—	—
54	2 (1.74)	—	—	—
56	5 (4.35)	—	—	—
58	7 (6.09)	—	—	2 (14.29)
59	1 (0.87)	1 (12.5)	—	2 (14.29)
61	1 (0.87)	—	—	—
62	3 (2.61)	—	—	—
66	5 (4.35)	—	—	—
68	2 (1.74)	—	—	—
70	11 (9.57)	—	—	1 (7.14)
74	1 (0.87)	—	—	—
87	1 (0.87)	—	—	—
L1AE	1 (0.87)	—	—	—
MM4	1 (0.87)	—	—	—
MM8	1 (0.87)	—	—	—
PCR result negative	25 (21.74)	—	—	—

Abbreviations: HPV=human papillomavirus; HSIL=high-grade squamous intraepithelial lesion.
